# Open-source satellite enumeration to map households: planning and targeting indoor residual spraying for malaria

**DOI:** 10.1186/s12936-015-0831-z

**Published:** 2015-09-17

**Authors:** Aniset Kamanga, Silvia Renn, Derek Pollard, Daniel J Bridges, Brian Chirwa, Jessie Pinchoff, David A Larsen, Anna M Winters

**Affiliations:** Akros, Cresta Golfview Grounds, Great East Road, Unit 5, Lusaka, Zambia; Presidents Malaria Initiative, Africa Indoor Residual Spray Project, Bethesda, USA; John Hopkins Bloomberg School of Public Health, Baltimore, MD USA; Syracuse University Department of Public Health, Food Studies and Nutrition, Syracuse, NY USA; University of Montana School of Public and Community Health Sciences, Missoula, MT USA

**Keywords:** Malaria, Indoor residual spraying, Google Earth, Enumerate, Structure, mSpray, Ground-truth

## Abstract

**Background:**

Defining the number and location of sprayable structures (houses) is foundational to plan and monitor indoor residual spray (IRS) implementation, a primary intervention used to control the transmission of malaria. Only by mapping the location and type of all sprayable structures can IRS operations be planned, estimates of spray coverage determined, and targeted delivery of IRS to specific locations be achieved. Previously, field-based enumeration has been used to guide IRS campaigns, however, this approach is costly, time-consuming and difficult to scale. As a result, field-based enumeration typically fails to map all structures in a given area, making estimations less reliable and reducing the enumerated coverage.

**Methods:**

Using open source satellite imagery and Geographic Information System software, satellite enumeration was conducted to guide IRS operations in 15 districts (91,302 km^2^) in northern Zambia during the 2014 spray season. Cost of satellite enumeration was compared to standard enumeration. Enumerated households were sampled to estimate sprayable surface area and wall type from the satellite enumeration using linear and logistic regression, respectively.

**Results:**

In comparison to the traditional field-based enumeration procedure, satellite-based enumeration was 22 times faster, and 10 times less costly. An estimated 98 % of the satellite enumerated buildings correctly classified roof type. Predicted surface area of each household correlated at a value of 0.91 with measured surface area of each household.

**Conclusion:**

For IRS campaigns, high quality and high coverage enumeration data aid in planning, through informed insecticide procurement. Through the identification of geographical areas and populations to target, enumeration data guide operations and assist monitoring and evaluation of IRS through the unbiased estimation of coverage achieved. Satellite enumeration represents a quick, cheap and accurate system to provide these data, and has potential applications beyond IRS for delivery of other targeted or non-targeted interventions (e.g. net distributions, mass drug administration, immunization campaigns, or even sampling frames for field studies).

## Background

Malaria is a disease caused by a parasite transmitted through mosquitoes of the *Anopheles* mosquitoes and is a leading cause of child mortality; in 2013, approximately 584,000 deaths were attributed to malaria, most of which were children [1]. An estimated 1.5 billion individuals throughout the globe are at risk of *Plasmodium falciparum* malaria transmission, the species which causes the most mortality, with the greatest number at risk on the African continent [2]. A primary intervention is the use of indoor residual spraying (IRS), which entails spraying an insecticide on the inside walls of households. Female *Anopheles* typically rest on the inside walls of houses after taking a blood meal, and with effective IRS those female Anopheles are killed. IRS thus reduces both the amount of mosquitoes circulating in a household and decreases the age of the population of mosquitoes, effectively reducing malaria transmission when coverage is sufficiently high [3].

Millions of dollars are spent across the southern African region every year on IRS to control malaria. In Zambia alone, the total IRS budget crested US 16 million to cover six high-risk provinces in 2015. The absence of household address systems and population counts in this region leads to uncertainty in estimating numbers of households needing to be sprayed as well as the amount of insecticide required during IRS planning sessions. Following implementation of IRS, uncertainty also exists in estimates of houses that are actually sprayed.

To date, IRS in Zambia is largely done in three phases. First, census records are consulted to estimate the number of households in each district or region targeted for IRS. Second, field-based enumeration teams travel through targeted districts to enumerate and locate households. Records are typically kept on paper, though some programmes have begun to transition to electronic-based enumerations. Third, IRS teams are mobilized and move through the targeted areas spraying households as they find them. The total process requires significant financial inputs in the first and second phases even before any insecticide is sprayed. Inaccuracies in the estimate of the number of houses and thus amount of insecticide to be sprayed leads to inefficient use of labor and funds, which can potentially lead to inadequate coverage. Furthermore, there has been no validation check that the ground-based teams effectively enumerate the entire targeted areas, an important consideration given the rural nature of the region.

Advances in the availability and resolution of satellite imagery facilitate the use of remotely sensed data to enumerate areas. This paper relates the development of an effective, efficient, low-cost and scalable methodology to enumerate houses in 15 districts of Luapula and Central Province, Zambia in preparation for the 2014 spray season using freely available remotely sensed satellite imagery and freely available software.

## Methods

### Study area

**Fig. 1:**
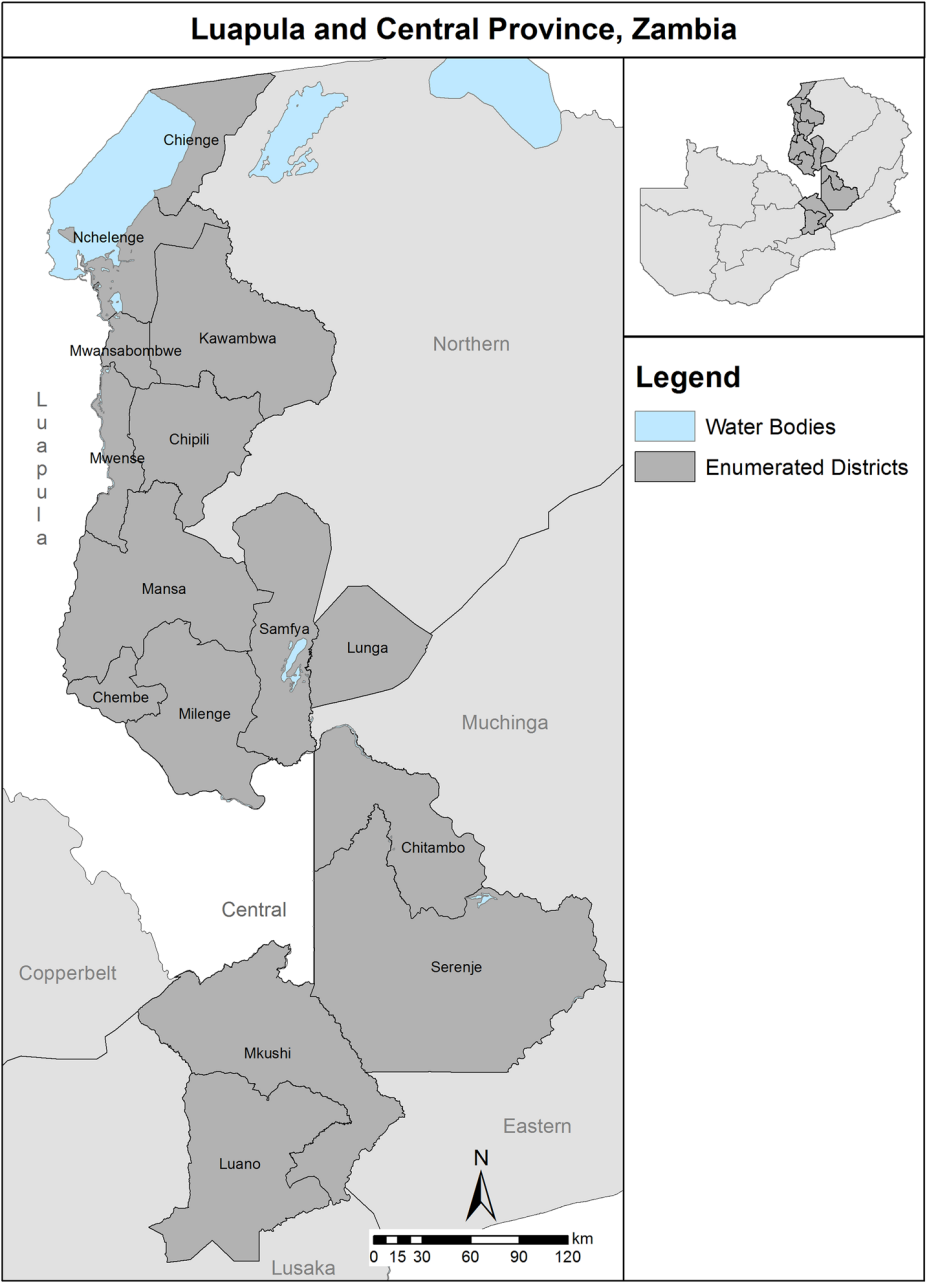
The study area included 15 districts (*dark grey*) of Luapula and Central Provinces of Zambia.

The study area included 15 districts of Luapula and Central Provinces of Zambia (Fig. 1). Luapula Province is divided into 11 districts with most of the land being surrounded by bodies of water such as lakes, rivers, and swamps. Central Province is divided into seven districts with the majority of the area covered by grassland and woodland. The population (de facto) of Luapula and Central Provinces are estimated at 938,000 and 1,246,000 respectively [4]. The provinces are largely rural (Luapula, 81 % rural; Central, 75 % rural); fishing and agriculture are the main sources of economic activity. Settlement patterns are largely clustered around roads and waterways.

### Household enumeration

A team of nine recent University graduates with little or no experience in using Geographic Information System (GIS) were given a 2-day GIS training which focused on Google Earth and Quantum GIS (QGIS) version 1.8.0, a freely available and open source GIS software package (http://www.qgis.org). The training focused on image interpretation, digitization and creation of vector data. Google Hybrid and Bing imagery plugins were loaded to complement each other during the screen digitization of the study area. Google Hybrid imagery was consulted first and where Google Hybrid imagery had missing or low resolution, Bing imagery was applied. Most images, by both providers, were taken between 2011 and 2013. For this reason the images correlated well in terms of visible structures. However, the location of the structures often varied for up to five metres between the Bing and Google images. Considering that most GPS devices have similar or higher discrepancies, this was not considered to be a hindering factor. Interestingly, once the enumeration neared completion, Google updated many of their images to 2014/2015 data. It can be assumed that Google updates images based on use/load of images.

**Fig. 2:**
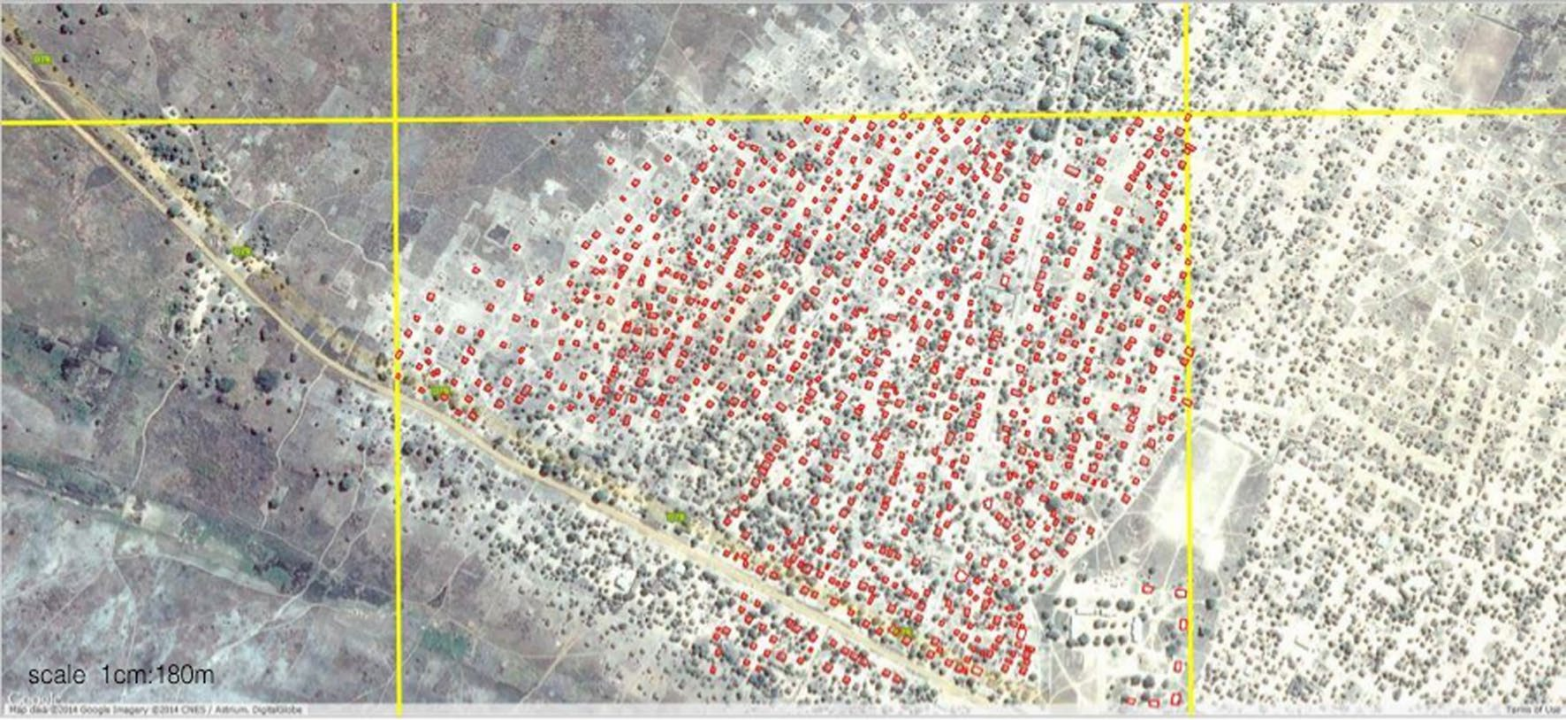
GIS supervisors divided the area into 1 km^2^ grid cells (*yellow lines* demarcate grid) and enumerators digitized structures within each grid cell by tracing a polygon (*red color*) around the outline of the roof of each structure. As an example of the process, all structures within the grid cell in the middle of the figure had been digitized; structures in all other cells had not yet been digitized.

The enumeration was conducted using an evolving methodology. First, GIS supervisors overlayed a grid with 1 km^2^ cells throughout the provinces to monitor the progression of the data capture (Fig. 2). The GIS technicians then mapped structures at the scale of 1:1,500 m through a screen digitization process. During the process, the GIS technicians traced a polygon around the outline of the roof of each structure. They were instructed to distinguish between thatched and non-thatched roofs in the associated attribute table. Smaller structures measuring <9 m^2^ were excluded in the digitization as these were not likely to be sleeping structures but rather toilets or granaries which are not sprayable structures according to national policy. Only sleeping structures are targeted with IRS. Larger structures were also excluded (buildings larger than 330 m^2^), as these were likely commercial or community-based buildings such as churches or warehouses and therefore not containing any sleeping spaces. As the supervisors and technicians proceeded, a more efficient workflow was developed. The GIS supervisors first assessed the study area and identified target areas with settlements of at least ten structures within each grid to be mapped and then conveyed these areas to the enumeration team for digitization (Fig. 2).

### Estimating sprayable surface area and wall type from satellite enumeration

A two-stage cluster sample of 125 randomly selected structures from four districts within the study area was conducted to develop a model predicting sprayable surface area from the satellite enumeration. The survey collected the following measures: type of roof of each structure, the amount of sprayable surface area in each house, and whether the house had smooth or porous walls. The captured data was used to test the ability of the satellite-based enumeration process to predict the sprayable surface area at the household level as well as whether or not the household had smooth wall surfaces. These data were necessary to ensure that IRS could be planned for based on variables easily identified through satellite imagery.

The total sample size of 125 houses was divided into 75 model fitting households and 50 model testing households. Three predictive regression models were conducted among the 75 model fitting households. First linear regression predicted the number of rooms in each house from the roof are of the enumerated structure (footprint) digitized from satellite imagery and the composition of the roof being either thatch or non-thatched as determined from satellite imagery. Second linear regression predicted the measured sprayable surface area as the outcome variable with the structure’s footprint, roof type and predicted number of rooms. Third the wall type (smooth or roof) was predicted from the structure footprint and the roof type. For the third model, three structures in the model fitting households had both rough and smooth walls and so were excluded from the predictive regression. In order to facilitate a linear relationship, the footprint variable and the outcome of sprayable surface area were log transformed for all three models. Accuracy of predicting sprayable surface area was assessed by applying coefficients from model fitting regression to the model testing data and estimating the correlation between measured sprayable surface area and predicted sprayable surface area. A receiver operating characteristic (ROC) curve defined sensitivity and specificity of correctly identifying the wall type in the model testing data using the predicted values. QGIS was used for map making [5]; R version 3.1.0 was used for all analyses; the pROC package was used to generate the ROC curves [6, 7].

#### Cost comparison of field versus satellite enumeration methods

A comparative cost analysis was conducted reviewing costs from traditional field-based enumeration with satellite-enumeration practices. Budgets from previous field-based enumeration exercises were consulted and compared with the budget for satellite-based enumeration. The metric of houses enumerated per day was used to compare the two methods and associated costs (field costs, travel, person-days) were also considered in the comparison.

## Results

### Household enumeration

The nine GIS technicians enumerated 270,000 structures across a total area of 91,302 km^2^ within a period of 22 days. On average, each technician was able to enumerate 1,364 structures per day (range 1,150–1,500).

### Model outcomes to estimate sprayable surface area and wall type from satellite enumeration

All structures sampled on the satellite imagery were successfully found in the field. The measured sprayable surface area among sampled structures ranged from 18.5 to 580.5 m^2^ with an interquartile range of 61.6–224.8 m^2^. Mean and median surface areas for non-thatched houses (typically steel-roofs) measured at 203.1 and 187.5 m^2^, respectively. Mean and median surface areas for thatched houses were measured at 68.3 and 52.2 m^2^, respectively.

### Predicting number of rooms per household

The log-transformed footprint (area) of the roof predicted the number of rooms with an R-squared of 0.76, *P* < 0.001 (Table 1); the type of roof added no explanatory power to the model. The difference in predicted number of rooms and measured number of rooms was typically <1 with a few outliers.

**Table 1:** Multivariate linear regression results predicting number of rooms per household, surface area of households and smooth wall surface within households

Predictor	Coefficient	Standard error	*P*-value
Predicting number of rooms per household^a^			
Intercept	−2.186	0.281	<0.001
Log-transformed area of roof	0.834	0.063	<0.001
Thatched roof (non-thatched is reference)	−0.005	0.071	0.948
Predicting surface area of households^b^			
Intercept	−0.719	0.918	0.436
Log-transformed area of roof	1.421	0.277	<0.001
Predicted number of rooms	−0.089	0.068	0.191
Thatched roof (non-thatched is reference)	−0.371	0.109	0.001
Predicting smooth wall surface within households^c^			
Intercept	−2.040	0.843	0.016
Area of roof	0.021	0.009	0.015
Thatched roof (non-thatched is reference)	−2.300	1.107	0.038

^a^N = 75, R^2^ = 0.758; ^b^N = 75, R^2^ = 0.751; ^c^N = 72.

### Predicting surface area of households

The log-transformed footprint (area) of the roof, predicted number of rooms, and roof composition (thatch versus non-thatch) were all associated with the surface area of the walls in bivariate analyses. Together they accounted for 75.8 % of the variation in surface area of the walls in the model fitting households (Table 1). Residual differences between predicted surface area of walls and measured surface area of walls among the model testing houses were typically small; correlation between measured and predicted surface area of walls among the 50 model testing households was 0.91. There was greater variation in the residuals of non-thatched roof households than in the residuals of thatched-roof households.

### Predicted wall type

Smooth wall surfaces were found in 40 (32 %) of the households sampled and rough wall surfaces were found in 80 (64 %) of the households sampled. Five households (4 %; three in the model fitting data and two in the model testing data) had both rough and smooth walls in different part of the house and were excluded from further analysis. Households with non-thatched roofs (steel) were 12 times more likely than households with thatched roofs to have smooth walled surfaces [prevalence ratio = 12.23, 95 % confidence interval (CI) = 2.76–54.22]. Houses with larger roofs were also more likely to have smooth walls compared to rough wall surfaces; mean roof area was 58.5 m^2^ for houses with rough wall surfaces and 113 m^2^ for houses with smooth wall surfaces (*t* test = −5.75, *P* < 0.001).

**Fig. 3:**
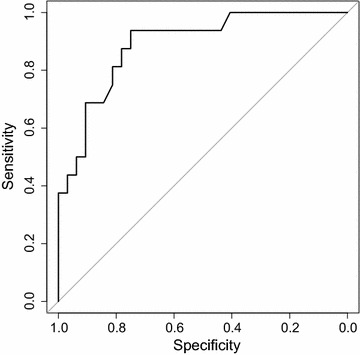
Receiver operator characteristic curve showing sensitivity and specificity of correctly identifying smooth or rough wall surfaces using different probability cutpoints from simple logistic regression.

The area of the roof and type of roof were both significantly associated with having smooth walls in logistic regression (Table 1). Predicted number of rooms did not improve the model and so was excluded from the logistic regression. From the 50 model testing households, the receiver operator characteristic curve shows good sensitivity and specificity for correctly identifying the wall type (Fig. 3). The predicted probability of having smooth walls with the largest area under the curve was 0.22, which gave a sensitivity of 93.8 % and a specificity of 75.0 %.

### Cost analysis

Given the same geographical scope, satellite enumeration was approximately ten times less expensive compared to field-based methods. Primary cost-drivers for field-based enumeration included staff costs (meal allowance and per diem for enumerators while in the field) followed by transport and supervision costs (Table 2). Field-based enumeration of one district was estimated at $12,062. In comparison, the cost to enumerate one district using a satellite-based approach was $1,177 with the primary cost driver also enumerator staff, in this case university-trained students who had recently graduated. No costs for fieldwork (fuel, transport, meal and per diems for staff) were necessary for the satellite-based process. All computer work was done at a local hired venue which did add a minimal cost, but included bandwidth necessary to complete the satellite-based process. Overall, in comparison to the traditional field-based enumeration procedure, satellite-based enumeration was faster, more efficient and less costly. On average, field-based enumeration teams are able to identify 60–70 structures per day (personal communication, Brian Chirwa, AfricaAIRS). In contrast, satellite-based enumeration mapped approximately 1,300 structures in a day, making satellite enumeration approximately 22 times faster. Due to remote nature of the districts covered, significant staffing and field logistics costs are saved through satellite enumeration. First, structures are visible from the ‘birds eye view’ and may be enumerated quickly through the use of satellite imagery versus requiring a team to travel large distances to remote areas and over difficult terrain, which likely misses structures considering the rural village context.

**Table 2:** In comparison to field-based enumeration, the satellite enumeration process was ten times less costly

Activity	Cost ($USD)	
	Field	Satellite
Fuel	541	0
Transport	2,143	0
Enumerators^a^	7,143	833
Supervisors^a^	1,786	29
GPS cost: 10 % contribution^b^	300	0
Data cleaning	150	48
Training venue hire	0	267
TOTAL per district	12,062	1,177

Costs are per district (average) and cost drivers are listed under ‘activity’.

^a^Including food and per diems as appropriate.

^b^GPS units were cost-shared amongst other projects.

## Discussion

In this study, use of satellite imagery for enumeration of structures was investigated for the planning and monitoring of IRS campaigns for malaria prevention and control. Previous studies have used satellite imagery and digitization for estimating population, creating sampling frames and building point feature class databases, however these studies did not use open-source imagery. Other studies that have used open-source imagery, did not use open-source GIS to process the imagery [8–13]. This study appears to be the first to use both freely available imagery and open-source GIS to conduct satellite enumeration. The expansion of freely available satellite imagery coupled with advancements in open-source GIS makes the methodology outlined herein applicable to various health sectors including estimating denominators for mass drug administration campaigns, net distributions, estimating coverage for IRS campaigns, estimating population size and creating sampling frames. The satellite enumeration methodology was found to be extremely cost-effective and efficient. Each technician was able to enumerate far more structures per day than an entire team of ground-based enumerators without the need for transport and field costs.

Aspects of households which are observable from satellite imagery (roof type and digitized surface area) were significantly associated with the type of wall surface, number of rooms and total sprayable wall surface area, characteristics important to consider for the procurement of insecticide commodities for IRS campaigns. This finding further identified the satellite enumeration methodology as an extremely useful and efficient tool for planning IRS campaigns.

Despite the advantage of being efficient, low-cost and scalable, various challenges were encountered during the satellite enumeration. In this study we ignored structures which had a roof surface area less than 9 m^2^ as these were most likely to be latrines, chicken coops or granaries which are not considered sprayable by the Government of the Republic of Zambia (GRZ). However, some animal shelters larger than 9 m^2^ could not be easily distinguished from structures where people slept. Also, it was difficult to isolate buildings which had contiguous roofing to determine if these were separate sleeping spaces, a problem which was also observed in a similar study [8]. Unfortunately, the enumeration accuracy was not assessed in terms of enumerating the correct (sprayable) structures, however, a study in Malawi found that fewer than 5 % of enumerated structures were not households but rather separate kitchens, stores or animal pens [11]. In a separate study in Zambia, only 6 (1 %) of 750 randomly selected sampled structures enumerated by satellite were not found [12]. A similar level of accuracy is likely for the methodology presented herein in its ability to rely on satellite imagery to build maps of sprayable (enumerated) structures and for those enumerated structures to contain sleeping spaces.

From observations during IRS field operations, two factors are suspected to affect the accuracy of the satellite enumeration. First, publicly available satellite imagery is between 1 and 3 years old and hence satellite enumerations do not account for population growth. Second, in some cases, canopy cover may obstruct the view of rooftops and making the satellite enumeration process more difficult. Cloud cover was not a significant impediment during enumeration as we were able to switch between Google Hybrid and Bing satellite imagery, utilizing the most cloud free image available. Further studies are planned to determine the extent these two factors affect the accuracy of the satellite enumeration.

## Conclusions

The satellite enumeration methodology described here represents a highly cost effective, scalable and efficient system for enumerating structures and describing their characteristics, e.g., footprint, roof type, wall area. Satellite enumeration data provides the foundation for determining an unbiased estimated of spray coverage during campaigns ensuring IRS impact is maximal. This system is superior to traditional ground enumeration, and should be considered for other applications requiring spatial targeting or planning e.g. mass drug administration, net distributions and for general sampling purposes.

## Abbreviations

IRS: indoor residual spraying
GRZ: Government of the Republic of Zambia
MOH: Ministry of Health
MCDMCH: Ministry of Community Development Mother and Child Health
GE: Google Earth

## References

[1] World Health Organization (2015) Malaria Fact Sheet. http://www.who.int/mediacentre/factsheets/fs094/en/. Accessed 29 July 2015

[2] Gething PW, Patil AP, Smith DL, Guerra CA, Elyazar IR, Johnston GL, Tatem AJ, Hay SI (2011). A new world malaria map: *Plasmodium falciparum* endemicity in 2010. Malar J.

[3] Pluess B, Tanser FC, Lengeler C, Sharp BL (2010) Indoor residual spraying for preventing malaria. Cochrane Database Syst Rev (4):CD006657. doi:10.1002/14651858.CD006657.pub2 10.1002/14651858.CD006657.pub2 PMC6532743 20393950

[4] Republic of Zambia Central Statistical Office: 2010 Census of Population and Housing (2010) http://unstats.un.org/unsd/demographic/sources/census/2010_phc/Zambia/PreliminaryReport.pdf. Accessed 31 July 2015

[5] QGIS: QGIS Geographical Information System (2015) http://qgis.osgeo.org. Accessed 29 July 2015

[6] R Project for Statistical Computing (2015) R: a language and environment for statistical computing. http://wwwR-project.org. Accessed 29 July 2015

[7] Robin X, Turck N, Hainard A, Tiberti N (2011). pROC: an open-source package for R and S+ to analyze and compare ROC curves. BMC Bioinform.

[8] Mohammad A, Shahid R, Jin-Kyung P, Shamoon S, Rion O, Qamaruddin N (2004). Use of satellite imagery in constructing a household GIS database for health studies in Karachi, Pakistan. Int. J Health Geogr.

[9] Veljanovski T, Kanjir U, Pehani P, Ostir K, Kovacic P, Escalante B (2012). Object-based image analysis of VHR satellite imagery for population estimation in informal settlement Kibera-Nairobi, Kenya. Remote sensing–applications.

[10] Schmitt U, Sulzer W, Schardt M, Fritsch D, Englich M, Sester M (1998). Analysis of settlement structure by means of high-resolution satellite imagery. Between visions and applications.

[11] Escamilla V, Emch M, Dandalo L, Miller WC, Martinson F, Hoffman I (2014). Sampling at community level by using satellite imagery and geographical analysis. Bull World Health Organ.

[12] Lowther SA, Curriero FC, Shields T, Ahmed S, Monze M, Moss WJ (2009). Feasibility of satellite image-based sampling for a health survey among urban townships of Lusaka, Zambia. Trop Med Int Health.

[13] Checchi F, Stewart B, Palmer J, Grundy C (2013). Validity and feasibility of a satellite imagery-based method for rapid estimation of displaced populations. Int J Health Geogr.

